# Lectures on the Anatomy and Physiolgy of Vision, by Dr. B. Joy Jeffries, M. D., of Boston

**Published:** 1871-04

**Authors:** 


### Lectures on the Anatomy and Physiolgy of Vision, by Dr. B. Joy Jeffries, M. D., cf Boston.

A series of eighteen lectures will be delivered by Dr. B. Joy Jefferies, on the Anatomy and Physiology of Vision, at Boylston Hall, Cambridge, on Monday and Thursday afternoons, at 4 P. M., commencing April 10th. The lectures cannot fail of interesting medical men. Officers and members of any department of the University, graduates of this and other Colleges, and teachers of public schools have a right to admission. Other persons may be admitted to the course, on the payment of five dollars at the Steward's office.

